# Potential active compounds and molecular mechanism of Xuefu Zhuyu decoction for atherosclerosis, based on network pharmacology and molecular docking

**DOI:** 10.1097/MD.0000000000029654

**Published:** 2022-08-12

**Authors:** Yingyun Li, Boyu Liu, Lin Liu, Qing Xu, Quan Shen, Weikang Li, Jingshan Zhao

**Affiliations:** a Traditional Chinese Medicine Processing Technology Innovation Center of Hebei Province, College of Pharmacy, Hebei University of Traditional Chinese Medicine, China; b Hebei Key Laboratory of Traditional Chinese Medicine Research on Cardiocerebrovascular Disease, Hebei University of Traditional Chinese Medicine, China; c College of Basic Medical, Hebei University of Traditional Chinese Medicine, China.

**Keywords:** AKT1, atherosclerosis, molecular docking, network pharmacology, VEGFA, Xuefu Zhuyu decoction, β-carotene

## Abstract

To explore the potential active compounds and molecular mechanism of Xuefu Zhuyu decoction (XFZYD) in the treatment of atherosclerosis (AS) based on network pharmacology and molecular docking.

The effective components and action targets of XFZYD were screened by using TCMSP database. And then, the action targets of AS were collected by GeneCards database. The intersection targets between the effective components’ targets of XFZYD and AS-related action targets were used to construct PPI networks. GO and Kyoto Encyclopedia of Genes and Genomes enrichment analysis were performed on these intersection targets. Finally, molecular docking software was used to excavate the active compounds of the core targets VEGFA and AKT1.

We detected 225 active components of XFZYD, and found that quercetin, kaempferol, luteolin, naringenin, β-sitosterol, isorhamnetin, stigmasterol, baicalein, nobiletin, and β-carotene are the potential active compounds of XFZYD; STAT3, IL6, JUN, VEGFA, MAPK14, and AKT1 are the core target proteins of the active compounds, among which VEGFA and AKT1 are the key target proteins. PPI network results showed that β-carotene, quercetin, kaempferol, luteolin, and naringenin had higher degree values and more corresponding targets than other 5 active compounds and had the stable binding ability to regulatory proteins VEGFA and AKT1. The core components β-carotene, quercetin, kaempferol, and luteolin exerted their therapeutic effects on AS by acting on the key target proteins VEGFA and AKT1 to regulate fluid shear stress and the AGE-RAGE signaling pathway and IL-17 signaling pathway of diabetic complications of AS. The molecular docking results showed that VEGFA and AKT1 had great docking ability with the targeted active compounds, and β-carotene is the best.

The active components of XFZYD, including β -carotene, quercetin, kamanol, and luteolin, can act on VEGFA and AKT1. These active ingredients play a role in alleviating and treating AS by regulating fluid shear stress and participating in signaling pathways such AS AGE-RAGE of atherosclerosis and diabetes mellitus complicated with AS. β-carotene is a potential inhibitor of VEGFA and AKT1 and treats AS through antioxidant action.

## 1. Introduction

Atherosclerosis (AS) is a critical cause of many cardiovascular and neurological diseases.^[1]^ Statins are currently restricted in their use in the clinic due to liver and muscle damages,^[2]^ so it is of practical significance to find more safe and effective drugs to prevent AS. In Traditional Chinese Medicine (TCM ), AS belongs to the category of “pulse carbuncle” and “pulse paralysis,” which could affect the blood vessels. AS can result in cardiovascular disease such as strokes, myocardial infarction, and chest arthralgia. Blood stasis, according to ancient doctors, was astringent and stagnant, gathered heat and formed poison, and toxic evil was most likely to injure muscles and veins.^[3]^ A therapeutic treatment strategy involves improving circulation and reducing blood stasis.

The Xuefu Zhuyu decoction (XFZYD), created by Wang Qingren in the Qing Dynasty, is the therapy for blood stasis which including Persicae semen (Taoren), Carthami flos (Honghua), AngElicae sinensis radix (Danggui), Rehmanniae radix (Dihuang), Achyranthis bidentatae radix (Niuxi), Chuanxiong rhizome (Chuanxiong), Platycodonis radix (Jiegeng), Paeoniae radix rubra (Chishao), Aurantii fructus (Zhiqiao), and Bupleuri radix (Chaihu). It can improve blood flow, reduce blood congestion, boosts energy, and relieves pain.^[4]^ Clinical research reported that XFZYD can significantly improve microcirculation, expand microvessels, increase tissue perfusion, alleviates clinical symptoms, and so on.^[5–7]^ Many diseases, including myocardial fibrosis, AS, hypertension, unstable angina pectoris, and myocardial ischemia-reperfusion injury, have been treated using XFZYD.^[8,9]^ However, more research is needed into the material basis and molecular targets of multicomponent, multichannel, and multitarget XFZYD in the prevention and treatment of as to give an evidence-based foundation for clinical wide application and new drug development.

Network pharmacology has become a brilliant way of studying traditional Chinese medicine’s multicomponent and multitarget action mechanism. This method employs bioinformatics, system biology, and multidrug biology to integrate network analysis, which breaks down the multilevel and all-encompassing drug mechanism into its constituent parts.^[10,11]^ In this study, the network pharmacology method was combined with molecular docking technology to predict the therapeutic mechanism of multitarget and multilevel synergistic application of pharmacodynamic active components in XFZYD for the treatment of AS, which helps to clarify the molecular mechanism of XFZYD in the treatment of AS and improve the effectiveness of the drug.

## 2. Materials and Methods

### 2.1. Ethical issues and other conflicts of interest

GeneCards and PDH belong to public databases. The patients, animals, and protein structures involved in these databases have obtained ethical approval. These data have been maintained publicly available for any researchers to use. Thus, the relevant data could be downloaded gratuitously for users to do some research and publish relevant articles. Our study is based on the open-source data, so there are no ethical issues and other conflicts of interest.

### 2.2. Acquisition of XFZYD active ingredients

Using TCM System Pharmacology database and analysis platform (TCMSP, https://old.tcmsp-e.com/tcmsp.php), the compounds in XFZYD was obtained. The effective compounds were screened according to oral bioavailability ≥ 30% and drug-like ≥ 0.18. With the help of the UniProt database (https://www.uniprot.org), we converted the collected active components into gene names.

### 2.3. Acquisition of AS disease targets

AS disease targets were retrieved from the GeneCards database (https://www.genecards.org), search “AS.” We screened for AS disease targets to satisfy the Relevance score ≥ 3 of the disease set.

### 2.4. Construction and analysis of component-intersection target network

The related targets of XFZYD and the targets of AS were obtained. The intersection targets of the related targets of XFZYD and AS disease targets as well as the active compounds of XFZYD were introduced into Cytoscape 3.8.2 software, so as to construct “Components-AS-target” network, and then analyze and study the network.

### 2.5. PPI network building and key objective screening

Import the intersection target into the String database (https://string-db.org/), choose Homo Sapiens as the race, and set the degree of confidence to 0.9 (the degree of confidence score represents the degree of protein interaction). We put the PPI network node data from the string database into Cytoscape 3.8.2 for network topology analysis and visualization, and the top 15 nodes are chosen as the core target of XFZYD for the treatment of AS, using degree as the screening criterion point.

### 2.6. Go and KEGG enrichment analysis

Set the threshold value to *P* ≤ .5 using the R language’s “clusterprofiler” computer package, choose 3 modules for enrichment analysis: biological process (BP), molecular function (MF), and cell composition (CC), then visually analyze the top 10 entries in each module. The intersection targets were analyzed using the KOBAS 3.0 database (http://kobas.cbi.pku.edu.cn/) for Kyoto Encyclopedia of Genes and Genomes (KEGG) enrichment, and we chose the first 15 signal pathways as significant enrichment pathways.

### 2.7. Molecular docking

The core target protein, whose species is human and the protein crystal resolution is <3A, was obtained in the PDH database (http://www.rcsb.org/). The 3D structure of effective compounds and the position control’s target core protein was obtained from the PubChem database (https://pubchem.ncbi.nlm.nih.gov/). We download the 3D structure of active compounds in XFZYD targeting core protein and positive control in the PubChem database (https://pubchem.ncbi.nlm.nih.gov/). Utilize the Molecular Operating Environment (MOE v2019.0102 software) to optimize the target protein structure (dehydration and hydrogenation, energy optimization) and minimize the energy of the active compound, as well as perform molecular docking of the processed target protein and small molecule compounds. To verify the binding of the target protein and the target active chemical, RSMD is utilized as the accuracy parameter of the molecular docking model (RSMD ≤ 4 A is reliable, RSMD ≤ 2 A is accurate), and S (E refine, the unit is kcal/mol) is used as the binding free energy parameter.

## 3. Results

### 3.1. Obtain XFZYD and AS intersection target

**Figure 1.**
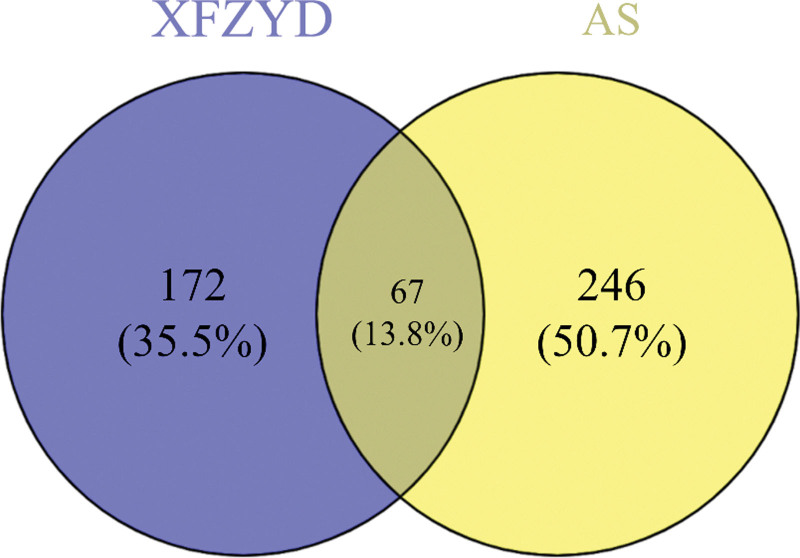
Venny map of XFZYD targets and AS targets. AS = atherosclerosis, XFZYD = Xuefu Zhuyu decoction.

Table 1 shows that there are 225 active components in total in XFZYD. Partial components are shown after comprehensive ranking according to oral bioavailability and drug-like. There were 239 XFZYD targets after the duplicate data were removed. There were 4681 action targets in the GeneCards database with “AS” as the keyword, of which 313 met the Relevance score ≥ 3 as AS’s action targets. Figure 1 demonstrates that XFZYD and AS have a total of 67 intersecting targets, including safflower, bupleurum, Achyranthes bidentata, and licorice, as indicated in Table 2.

**Table 1:** Main components of XFZYD

TCM	ID	MOL ID	Active ingredient	OB (%)	DL
Carthami flos	HH1	MOL002712	6-Hydroxykaempferol	62.13	0.27
	HH2	MOL002680	Flavoxanthin	60.41	0.56
	HH3	MOL002717	qt_Carthamone	51.03	0.20
	HH4	MOL002710	Pyrethrin II	48.36	0.35
	HH5	MOL000098	Quercetin	46.43	0.28
	HH6	MOL002721	Quercetagetin	45.01	0.31
	HH7	MOL000449	Stigmasterol	43.83	0.76
	HH8	MOL002695	Lignan	43.32	0.65
	HH9	MOL002707	Phytofluene	43.18	0.50
	HH10	MOL000422	Kaempferol	41.88	0.24
	HH11	MOL002776	Baicalin	40.12	0.75
	HH12	MOL002706	Phytoene	39.56	0.50
	HH13	MOL000953	CLR	37.87	0.68
	HH14	MOL002773	Beta-carotene	37.18	0.58
	HH15	MOL001771	Poriferast-5-en-3beta-ol	36.91	0.75
	HH16	MOL000358	Beta-sitosterol	36.91	0.75
	HH17	MOL000006	Luteolin	36.16	0.25
	HH18	MOL002698	Lupeol-palmitate	33.98	0.32
	HH19	MOL002714	Baicalein	33.52	0.21
	HH20	MOL002719	6-Hydroxynaringenin	33.23	0.24
Persicae semen	TR1	MOL001371	Populoside_qt	108.89	0.20
	TR2	MOL001351	Gibberellin A44	101.61	0.54
	TR3	MOL001348	Gibberellin 17	94.64	0.49
	TR4	MOL001353	GA60	93.17	0.53
	TR5	MOL001344	GA122-isolactone	88.11	0.54
	TR6	MOL001329	2,3-Didehydro GA77	88.08	0.53
	TR7	MOL001360	GA77	87.89	0.53
	TR8	MOL001340	GA120	84.85	0.45
	TR9	MOL001339	GA119	76.36	0.49
	TR10	MOL001358	Gibberellin 7	73.80	0.50
Paeoniae radix rubra	CS1	MOL001918	Paeoniflorgenone	87.59	0.37
	CS2	MOL001925	Paeoniflorin_qt	68.18	0.40
	CS3	MOL007016	Paeoniflorigenone	65.33	0.37
	CS4	MOL006996	1-o-beta-d-glucopyranosylpaeonisuffrone_qt	65.08	0.35
	CS5	MOL007022	EvofolinB	64.74	0.22
	CS6	MOL007018	9-ethyl-neo-paeoniaflorin A_qt	64.42	0.30
	CS7	MOL006992	(2R,3R)-4-methoxyl-distylin	59.98	0.30
	CS8	MOL007008	4-ethyl-paeoniflorin_qt	56.87	0.44
	CS9	MOL000492	(+)-Catechin	54.83	0.24
	CS10	MOL001924	Paeoniflorin	53.87	0.79
Chuanxiong rhizoma	CQ1	MOL000433	FA	68.96	0.71
	CQ2	MOL002140	Perlolyrine	65.95	0.27
	CQ3	MOL002151	Senkyunone	47.66	0.24
	CQ4	MOL002157	Wallichilide	42.31	0.71
	CQ5	MOL001494	Mandenol	42.00	0.19
	CQ6	MOL002135	Myricanone	40.60	0.51
	CQ7	MOL000359	Sitosterol	36.91	0.75
Achyranthis bidentatae radix	NX1	MOL000785	Palmatine	64.60	0.65
	NX2	MOL000098	Quercetin	46.43	0.28
	NX3	MOL012542	β-ecdysterone	44.23	0.82
	NX4	MOL000449	Stigmasterol	43.83	0.76
	NX5	MOL002897	Epiberberine	43.09	0.78
	NX6	MOL001006	Poriferasta-7,22E-dien-3beta-ol	42.98	0.76
	NX7	MOL004355	Spinasterol	42.98	0.76
Rehmanniae radix	SDH1	MOL000449	Stigmasterol	43.83	0.76
	SDH2	MOL000359	Sitosterol	36.91	0.75
AngEelicae sinensis radix	DG1	MOL000449	Stigmasterol	43.83	0.76
	DG2	MOL000358	Beta-sitosterol	36.91	0.75
Platycodonis radix	JG1	MOL004580	cis-Dihydroquercetin	66.44	0.27
	JG2	MOL005996	2-O-methyl-3―O-β-D-glucopyranosyl Platycogenate A	45.15	0.25
	JG3	MOL004355	Spinasterol	42.98	0.76
	JG4	MOL006070	Robinin	39.84	0.71
	JG5	MOL000006	Luteolin	36.16	0.25
	JG6	MOL001689	Acacetin	34.97	0.24
Aurantii fructus	ZK1	MOL002341	Hesperetin	70.31	0.27
	ZK2	MOL005828	Nobiletin	61.67	0.52
	ZK3	MOL004328	Naringenin	59.29	0.21
	ZK4	MOL013381	Marmin	38.23	0.31
	ZK5	MOL000358	Beta-sitosterol	36.91	0.75
Bupleuri radix	CH1	MOL004644	Sainfuran	79.91	0.23
	CH2	MOL013187	Cubebin	57.13	0.64
	CH3	MOL000354	Isorhamnetin	49.60	0.31
	CH4	MOL004609	Areapillin	48.96	0.41
	CH5	MOL004628	Octalupine	47.82	0.28
	CH6	MOL004624	Longikaurin A	47.72	0.53
	CH7	MOL000098	Quercetin	46.43	0.28
	CH8	MOL004653	(+)-Anomalin	46.06	0.66
	CH9	MOL000449	Stigmasterol	43.83	0.76
Glycyrthizae radix et rhizoma	GC1	MOL002311	Glycyrol	90.78	0.67
	GC2	MOL004904	Licopyranocoumarin	80.36	0.65
	GC3	MOL004891	Shinpterocarpin	80.30	0.73
	GC4	MOL005017	Phaseol	78.77	0.58
	GC5	MOL004841	Licochalcone B	76.76	0.19
	GC6	MOL004810	Glyasperin F	75.84	0.54
	GC7	MOL001484	Inermine	75.18	0.54
	GC8	MOL000500	Vestitol	74.66	0.21
	GC9	MOL005007	Glyasperins M	72.67	0.59

**Table 2:** Effective components and predicted targets number of traditional Chinese medicine

Name of traditional Chinese medicine	Quantity of active ingredients	Number of predicted targets	Common target with AS
Carthami flos	22	186	51
Persicae semen	23	35	5
Glycyrthizae radix et rhizoma	92	199	62
AngEelicae sinensis radix	2	40	5
Achyranthis bidentatae radix	20	363	79
Chuanxiong rhizoma	7	22	9
Platycodonis radix	7	63	19
Paeoniae radix rubra	29	80	18
Aurantii fructus	5	69	26
Bupleuri radix	17	166	50
Rehmanniae radix	2	26	3

### 3.2. Construction and analysis of component-intersection target network

**Figure 2.**
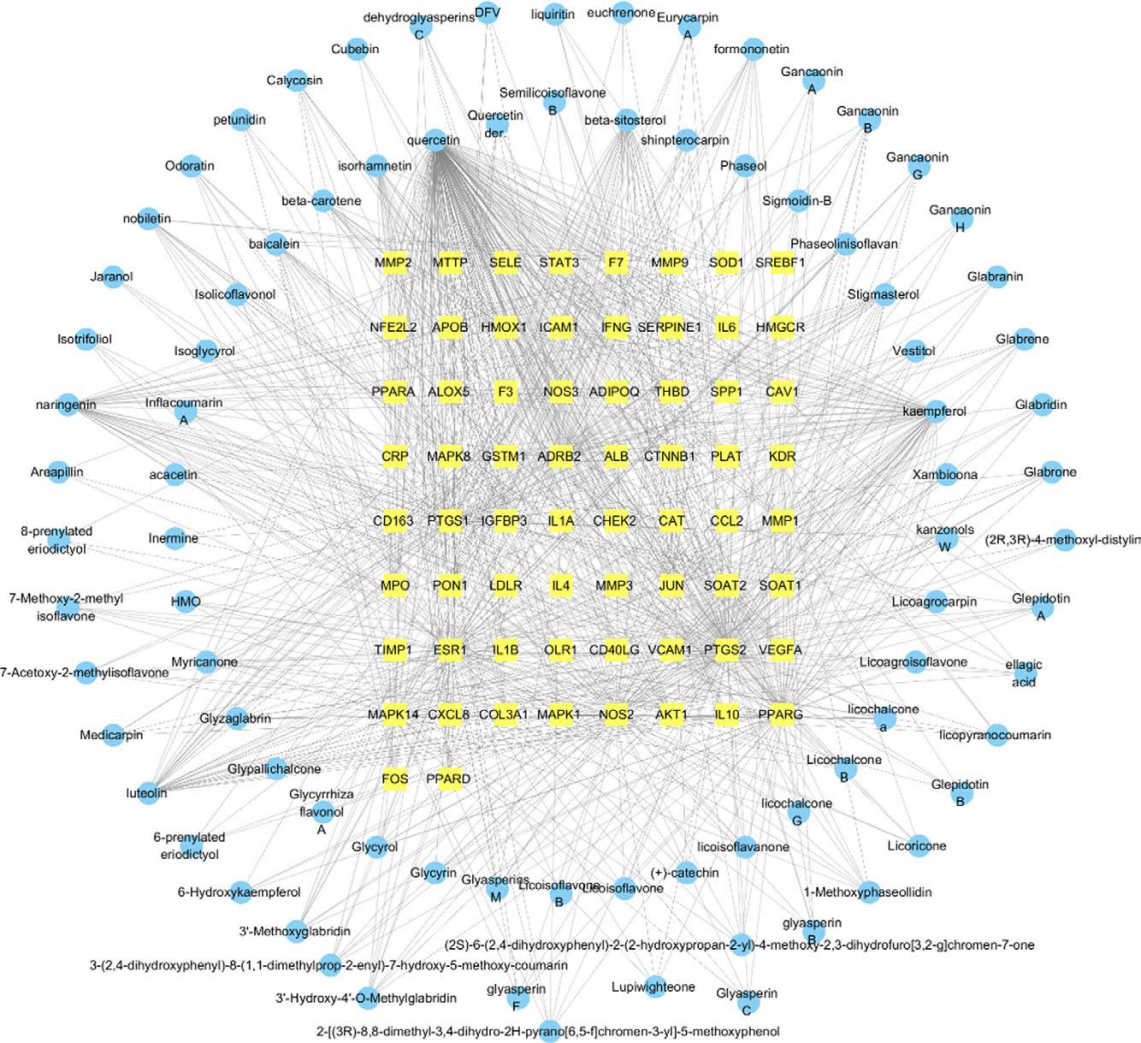
The active compound-AS target network of XFZYD. AS = atherosclerosis, XFZYD = Xuefu Zhuyu decoction.

The network diagram of traditional Chinese medicine component target interaction was generated and mapped using Cytoscape 3.8.2 software based on the active components and intersection genes in XFZYD. The drug’s active ingredient is represented by the outer circle, the intersection gene target is represented by the middle rectangular node, and the correlation between the active ingredient and the intersection target is represented by the edge in Figure 2. By the “active compounds—intersection targets” network of active compounds in the degree of value ranking shows that the more the greater the value of the corresponding target, top compounds with quercetin, kaempferol, luteolin, naringenin, β-sitosterol, isorhamnetin, stigmasterol, baicalein, nobiletin, and β-carotene (Table 3).

**Table 3:** Key components of XFZYD

Active ingredients	Degree	Source
Quercetin	184	Carthami flos, Bupleuri radix, Glycyrthizae radix et rhizome
Kaempferol	64	Carthami flos, Glycyrthizae radix et rhizome, Achyranthis bidentatae radix
Luteolin	34	Platycodonis radix
Naringenin	34	Aurantii fructus, Glycyrthizae radix et rhizoma
beta-sitosterol	30	AngEelicae sinensis radix, Carthami flos, Persicae semen, Paeoniae radix rubra, Aurantii fructus
Isorhamnetin	18	Bupleuri radix, Glycyrthizae radix et rhizome, Achyranthis bidentatae radix
Stigmasterol	15	AngEelicae sinensis radix, Carthami flos, Bupleuri radix, Paeoniae radix rubra, Rehmanniae radix, Achyranthis bidentatae radix
Baicalein	14	Carthami flos, Paeoniae radix rubra, Achyranthis bidentatae radix
Nobiletin	12	Aurantii fructus
beta-carotene	11	Carthami flos

### 3.3. Construction of the PPI network and key node screening

**Figure 3.**
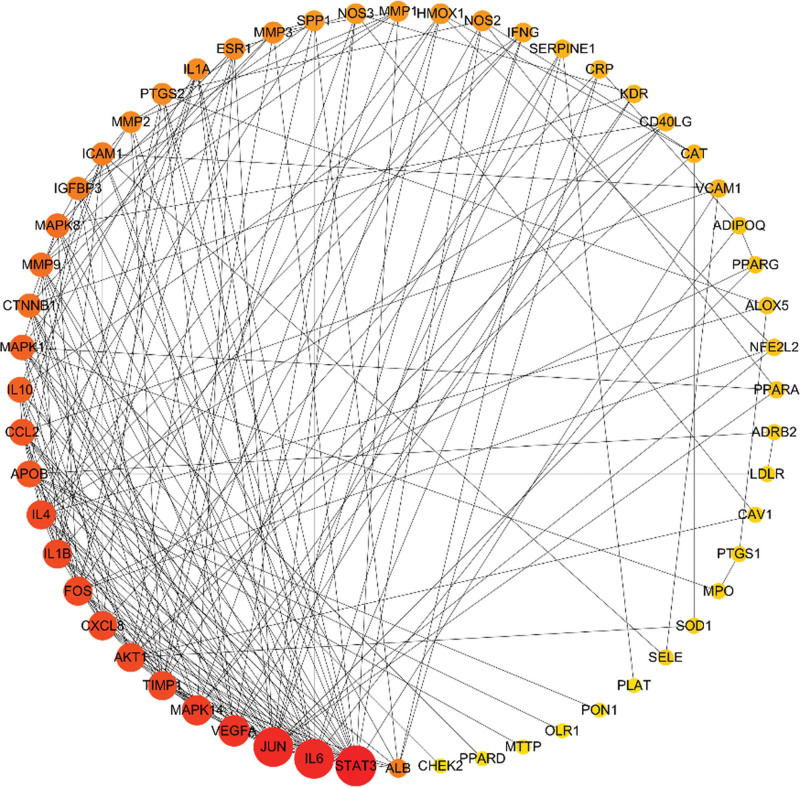
Protein-protein interactions network of AS targets of XFZYD. AS = atherosclerosis, XFZYD = Xuefu Zhuyu decoction.

**Figure 4.**
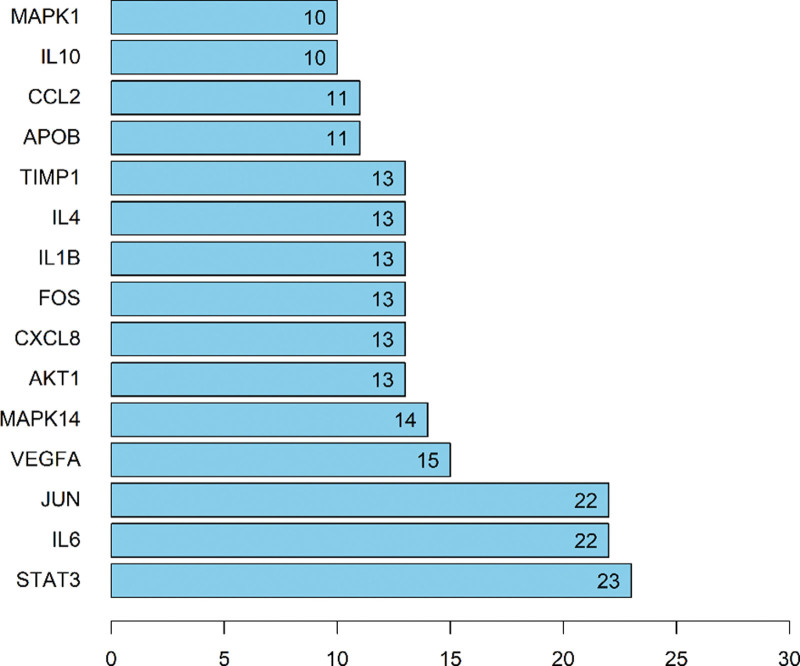
The top 20 of core target proteins in PPI network.

We imported the intersection targets into the string database for analysis and set the confidence level to ≥9. Then we used Cytoscape software to build the PPI network and analyze the network topology. The PPI network includes 67 nodes and 1076 edges, with a node degree of 32.1 on average (shown in Fig. 3). The darker the node color is, the larger the diameter is, representing the larger Degree value in Figure 3. Based on the PPI network’s degree values, the top 6-core target proteins were STAT3, IL6, Jun, VEGFA, Mapk14, and AKT1 (Fig. 4).

### 3.4. Go and KEGG enrichment analysis

**Figure 5.**
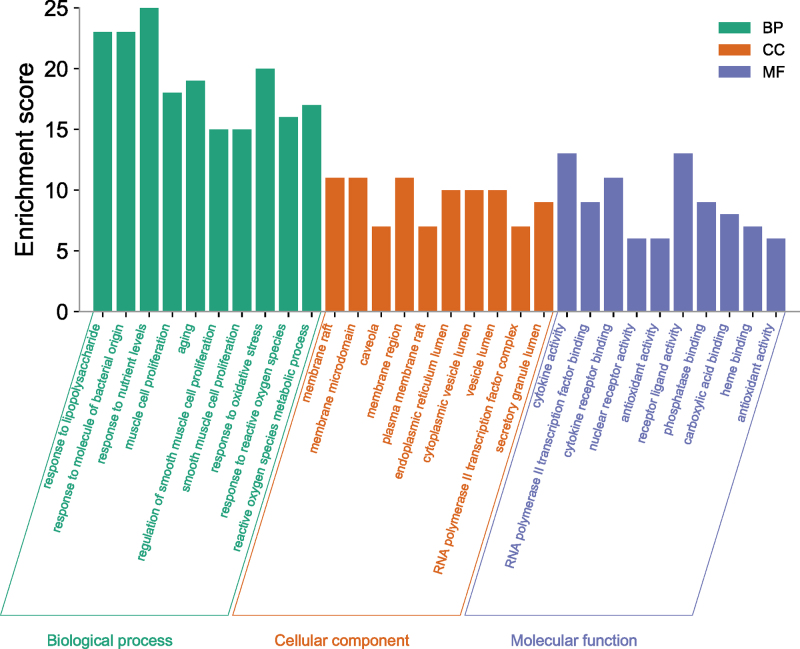
GO functional analysis of AS targets of XFZYD. AS = atherosclerosis, XFZYD = Xuefu Zhuyu decoction.

The results of GO enrichment analysis revealed that GO functional enrichment analysis yielded 1761 GO entries, including 1636 BP entries, 43 CC entries, and 82 MF-related entries. BP is mainly related to lipopolysaccharide, nutrient level and oxidative stress, and oxidative stress. CC is mainly connected with membrane rafts, collagen-containing extracellular matrix, secretory granule cavities, platelet α particle cavities, and similar structures. Cytokine activity, RNA polymerase II transcription factor binding, and cytokine receptor binding are all associated with MF (Fig. 5).

**Figure 6.**
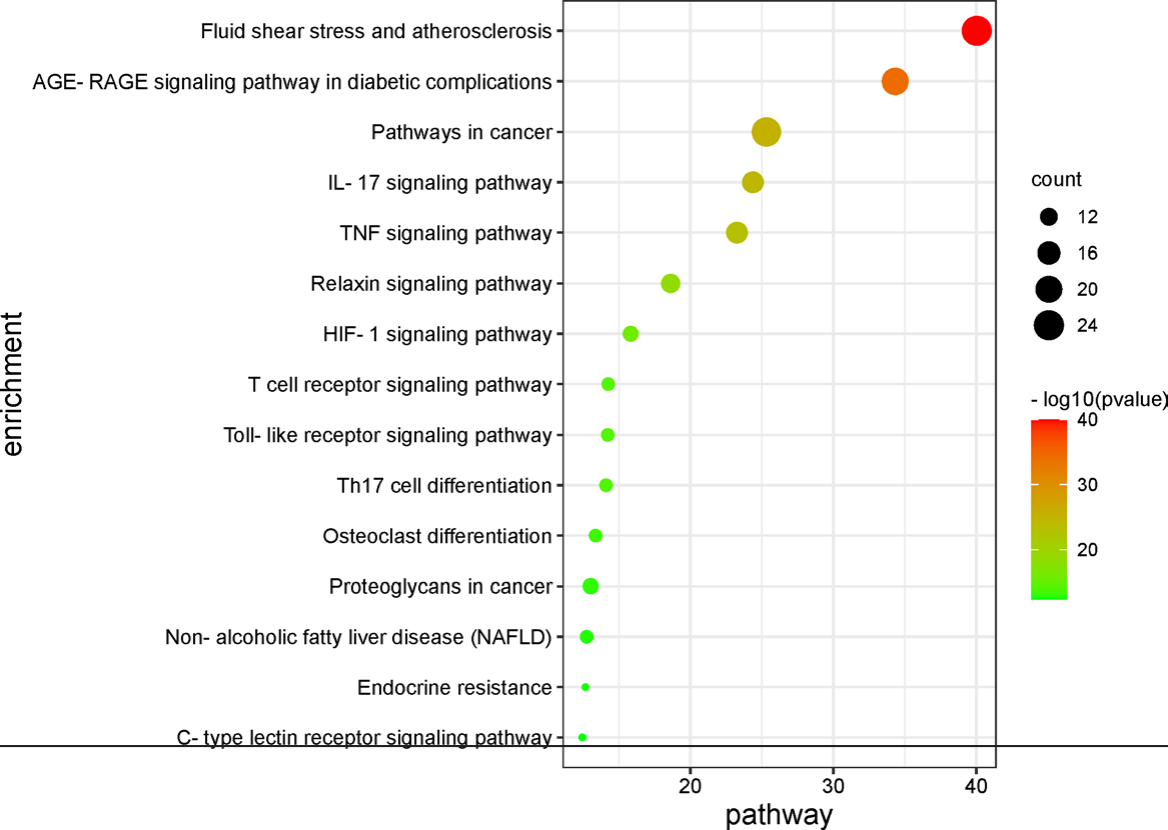
The top 15 of KEGG enrichment analysis of AS of XFZYD. KEGG = Kyoto Encyclopedia of Genes and Genomes, XFZYD = Xuefu Zhuyu decoction.

**Figure 7.**
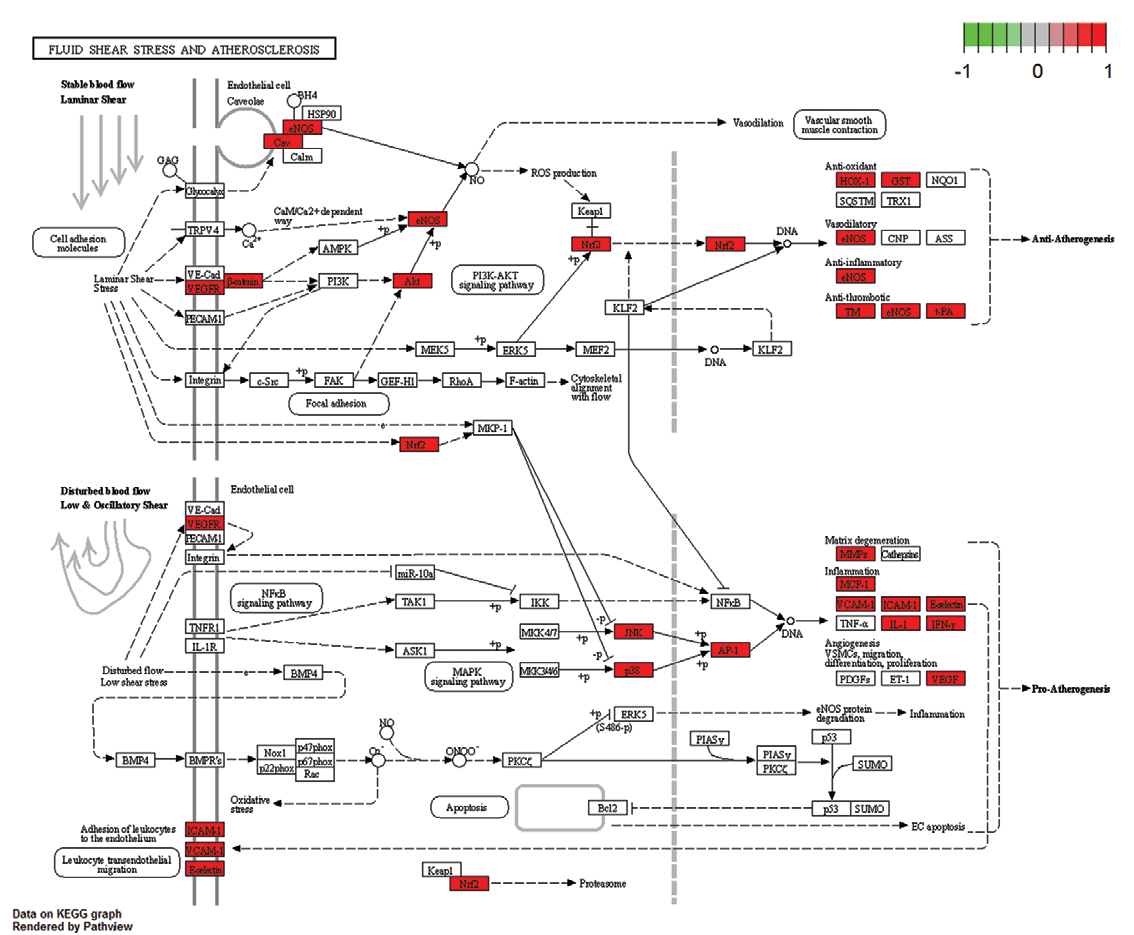
Fluid shear stress and AS of XFZYD. AS = atherosclerosis, XFZYD = Xuefu Zhuyu decoction.

**Figure 8.**
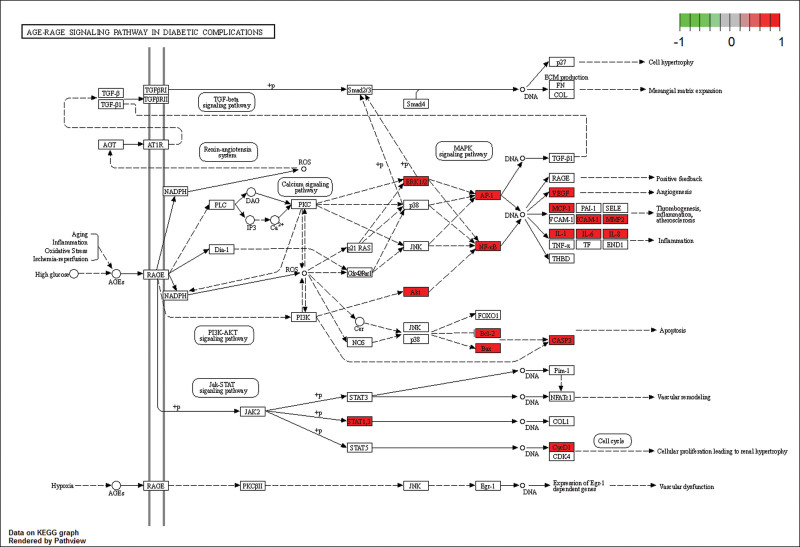
AGE-RAGE signaling pathway of XFZYD. XFZYD = Xuefu Zhuyu decoction.

KEGG pathway enrichment analysis was performed on 67 intersection targets using KOBAS 3.0 database, then draw the bubble diagram by the top 15 signal pathways. The color of the bubble denotes the degree of enrichment, while the size of the bubble represents the number of enriched genes (Fig. 6). The results indicate that XFZYD may influence the occurrence and progression of AS via fluid shear stress, and signaling pathways such as AGE-RAGE, TNF, and IL-17. The results show that XFZYD might regulate fluid shear stress and atherosclerosis, AGE-RAGE signaling pathway, TNF signaling pathway, and IL-17 signaling pathway to influence the development of AS (Figs. 7 and 8).

### 3.5. Molecular docking

**Figure 9.**
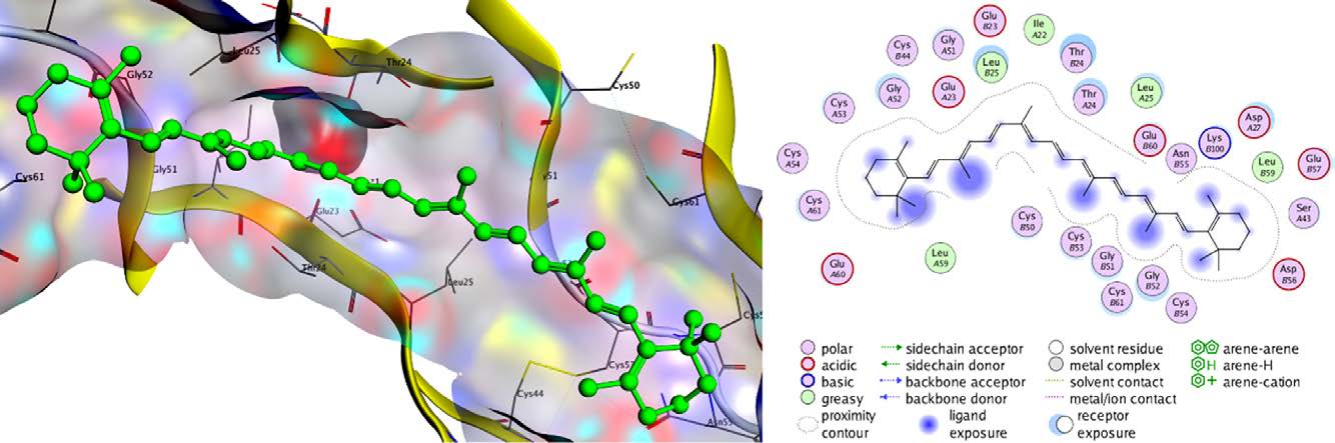
Docking pattern of β-carotene and VEGFA.

**Figure 10.**
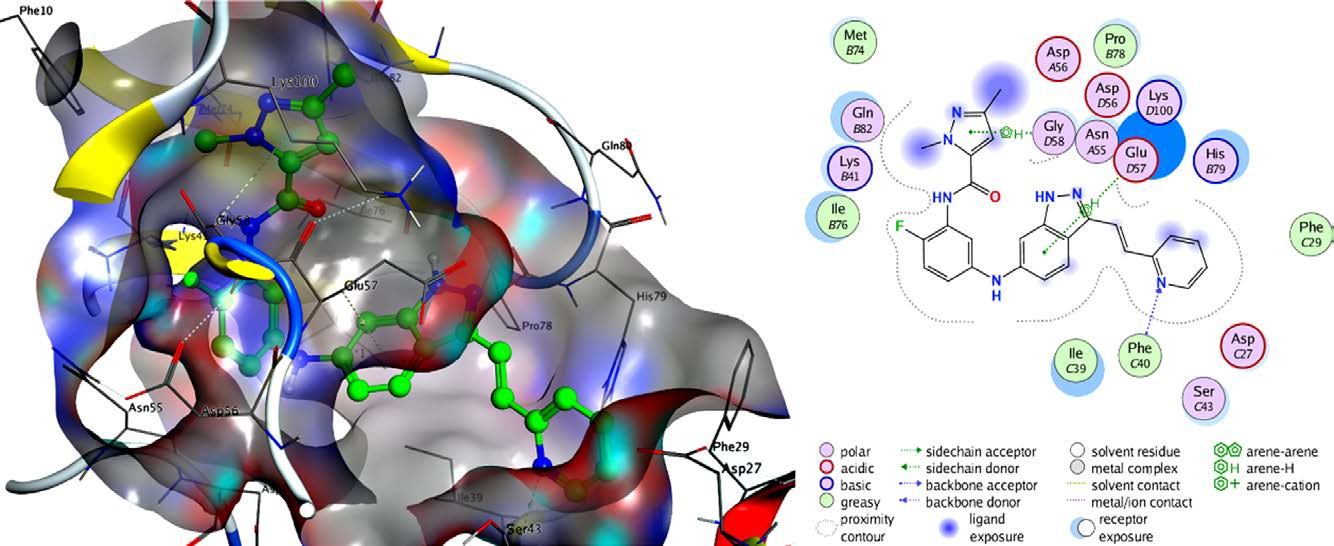
Docking pattern of AG-13958 and VEGFA.

**Figure 11.**
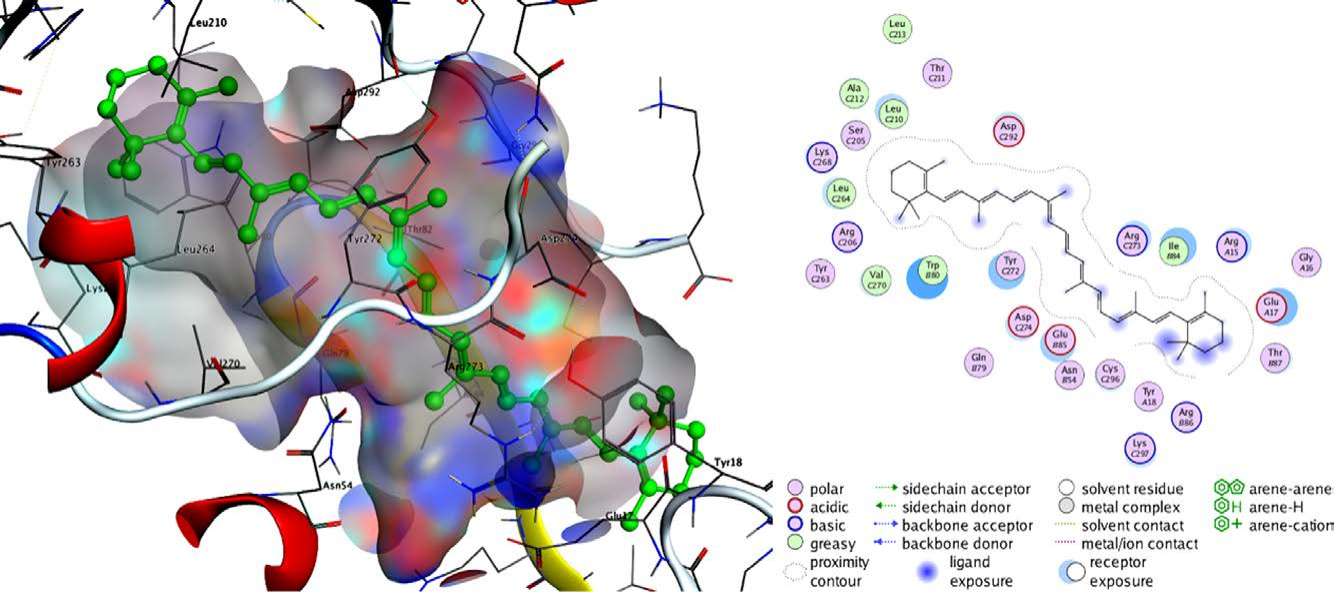
Docking pattern of β-carotene and AKT1.

**Figure 12.**
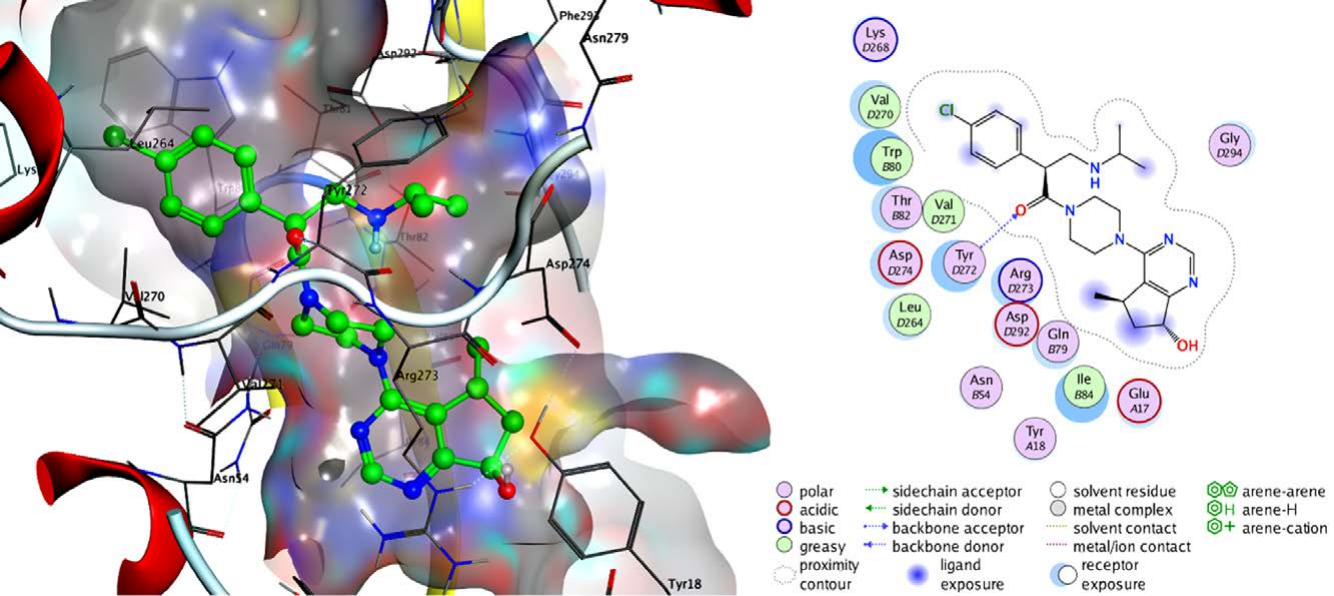
Docking pattern of ipatasertib and AKT1.

The docking results in Table 4 indicate that active drugs targeting the VEGFA and AKT1 proteins have a high affinity for their targets (S < –6 kcal/mol) and an accurate binding model (RMSD < 2). We chose positive controls (AG-13958 for VEGFA and ipatasertib for AKT1) as a reference. The binding of β-carotene, quercetin, kaempferol, luteolin, and naringin to VEGFA and AKT1 protein targets was more stable than the positive control, and β-carotene binding was the strongest (S < 8 kcal/mol) (Figs. 9–12).

**Table 4:** The molecular docking score of core target proteins with active compounds

Core target	PDBID	Targeted active compound	Source	S (kcal/mol)	RMSD
VEGFA	3QTK	β-Carotene	Carthami flos	–8.5126	1.7990
		Quercetin	Carthami flos	–6.4146	2.7384
			Bupleuri radix		
			Glycyrthizae radix et rhizoma		
			Achyranthis bidentatae radix		
		luteolin	Carthami flos	–6.1290	0.6845
			Platycodonis radix		
		Baicalein	Carthami flos	–6.1284	1.0777
			Paeoniae radix rubra		
			Achyranthis bidentatae radix		
		Ellagic acid	Paeoniae radix rubra	–6.1038	1.2106
		AG-13958	positive control	–7.6979	1.8274
		PTC-299		–7.1679	1.4264
		NM-3		–6.0531	1.8093
AKT1	6HHF	β-Carotene	Carthami flos	–9.6154	1.4225
		Quercetin	Carthami flos	–6.5852	1.8190
			Bupleuri radix		
			Glycyrthizae radix et rhizoma		
			Achyranthis bidentatae radix		
		Kaempferol	Carthami flos	–6.4700	1.2972
			Bupleuri radix		
			Glycyrthizae radix et rhizomaAchyranthis bidentatae radix		
		Baicalein	Carthami flos	–6.4625	0.7620
			Paeoniae radix rubra		
			Achyranthes bidentata Bl		
		Naringenin	Aurantii fructus	–6.3506	1.0781
			Glycyrthizae radix et rhizoma		
		luteolin	Carthami flos	–6.3010	1.1852
			Platycodonis radix		
		Ipatasertib	positive control	–8.4336	1.0894
		Capivasertib		–8.1244	1.3326
		Afuresertib		–7.7206	1.2655

## 4. Discussion

AS is a risk factor in the occurrence and development of cardiovascular and cerebrovascular diseases. So far, researchers have not been able to fully understand AS because its mechanism is related to multiple factors and the activation of many genes.^[12]^ With changes in nutrition and living standards, the incidence of AS is increasing year after year and is trending younger. Therefore, controlling the occurrence and progression of atherosclerotic is an urgent problem to be solved.

The prospective mechanism of XFZYD in the treatment of AS was explored using network pharmacology and molecular docking technologies in this study. Based on the active compounds-intersection targets network of XFZYD, we screened out quercetin, kaempferol, luteolin, naringin, β-sitosterol, isorhamnetin, stigmasterol, baicalein, nobiletin, and β-carotene, all of which could bind to key regulatory proteins. Studies have shown that quercetin can scavenge superoxide free radicals and reduce oxidative stress damage. It also shows anti-inflammatory and antiapoptotic activities. In addition, quercetin has cardiovascular protection.^[13,14]^ Luteolin, a compound in various plants, has many pharmacological actions, including anti-inflammatory, antitumor, antiviral, uric acid lowering, and cardiovascular disease prevention and treatment. It has been shown that luteolin, which works by modulating the phenotypic transition of macrophages, can block angiotensin-induced apoptosis.^[15]^ Kaempferol could be beneficial in the treatment of AS by lowering cholesterol levels, promoting antioxidant defenses, and improving vasculitis.^[16]^ β-carotene is the primary precursor of vitamin A and its metabolite retinoic acid. Studies have shown that the conversion of β-carotene to vitamin A delays the development of atherosclerosis by reducing hepatic lipid secretion in mice. β-carotene also can improve atherosclerosis caused by vitamin A deficiency.^[17,18]^ The results showed that XFZYD contained many active compounds and played a synergistic role in the treatment of AS.

STAT3, IL6, Jun, VEGFA, Mapk14, and Akt are the primary target proteins in the PPI network (Fig. 4). STAT3 is a member of the STAT family of proteins, which are involved in cell growth, apoptosis, carcinogenesis, and other life processes.^[19]^ Monte et al^[20]^ reported that inhibiting vascular endothelial growth factor (VEGF) expression in human umbilical vein endothelial cells in vitro by decreasing STAT3 phosphorylation; hence, boosting VEGF-induced endothelial cell migration and proliferation and delaying the formation of atherosclerotic plaque. It is reported that STAT3 can bind to the promoter region of the VEGF gene and regulate its transcription.^[21,22]^ When the inflammatory inducible factor IL6 binds to relevant receptors, it can facilitate signal transmission in cells, activate the JAK2/STAT3 signal pathway, and boost vascular smooth muscle cell proliferation. Inactivation of the c-Jun/AP-1 signaling pathway can effectively relieve AS in vivo and reduce the proliferation and migration of atherosclerotic vascular smooth muscle cells (VSMCs), according to Rongjing Ji.^[23]^ The findings show that the major active chemicals operate on a variety of target proteins to serve an overall regulatory effect.

The KEGG pathway enrichment analysis revealed that XFZYD inhibited AS via modulating the fluid shear stress, the AGE-RAGE signal pathway, the TNF signal pathway, and the IL-17 signal pathway (Fig. 6). AS and fluid shear stress are inextricably linked. The balance of shear force is strongly correlated with the integrity of the vascular intima, as evidenced by research. Atherosclerotic plaque development is positively correlated with low shear force in blood flow. Atherosclerotic plaque development and dispersion are mostly determined by the presence of low blood flow shear force.^[24]^ Through oxidative stress, the AGE-RAGE signaling pathway can exacerbate vascular damage. Vascular damage is closely relative to plaque development in AS. Further investigation showed the pathways involved in the treatment of AS by XFZYD are related to the VEGFA signaling pathway, AKT1 signaling pathway, and MAPK14 signaling pathway. Meanwhile, the pathways involved in the treatment of diabetes complicated by AS by XFZYD are related to the VEGFA signaling pathway, AKT1 signaling pathway, and CCL2 signaling pathway. VEGFA is a critical angiogenesis growth factor, promoting macrophage infiltration, endothelial cell proliferation and differentiation, and foam cell formation.^[25]^ AKT1 is a serine/threonine-protein kinase that, among other things, helps regulate vascular endothelium and VSMCs and plays a role in protecting the cardiovascular system.^[26,27]^

The results of fluid shear stress and atherosclerosis enrichment pathway (Fig. 7), AGE-RAGE signaling pathway (Fig. 8), and PPI core protein ranking (Fig. 4) showed that VEGFA and AKT1 are closely related to the development of AS, and maybe the key regulatory proteins of XFZYD in the treatment of AS. We screened several main active compounds in XFZYD, such as luteolin,naringin, β-sitosterol, isorhamnetin, stigmasterol, baicalin, and β-carotene (Table 4), based on the TCMSP database and “active compounds-intersection targets network.” These screened compounds may act on VEGFA and AKT1 protein, then we do molecular docking. The findings reveal that active drugs that target the proteins VEGFA and AKT1 have a strong bond ability (S < –6 kcal/mol) and binding model accuracy (RMSD < 2). β-carotene, quercetin, kaempferol, luteolin, and naringenin are active components in XFZYD (Table 3) and play a significant regulatory role in the “active compounds-intersection targets network” (Fig. 2). We selected the positive control as a reference to perform molecular bonding of β-carotene, quercetin, kaempferol, luteolin, and naringenin to VEGFA and AKT1 target protein. The results showed that these active compounds all have stable binding to VEGFA and AKT1 target protein (Figs. 9–12), among which β-carotene binding ability is the best (S < –8 kcal/mol).

The active compound—intersection target network, degree ranking of key active compounds, and molecular docking results of apple were comprehensively analyzed, we found that β-carotene, quercetin, kaempferol, luteolin, and naringenin not only have higher degree values and correspond to more targets, but also have good binding ability to key regulatory proteins VEGFA and AKT1, they may be active chemicals with the potential to treat AS. The above results reflected that XFZYD has the biological characteristics of multicomponents, multitargets, and multipathways. These components, targets, and pathways are woven into a network to treat atherosclerosis at a holistic level.

In summary, we studied the enrichment analysis of action targets of XFZYD in treating atherosclerosis, then carried out the network topology analysis of the TCM component-action target network diagram and protein interaction network diagram. We found that β-carotene, quercetin, kaempferol, and luteolin in Safflower can act on VEGFA and AKT1 and other targets. By regulating the shear stress of vascular fluid and participating in the regulation of AGE-RAGE and other signaling pathways in atherosclerosis and diabetes mellitus complicated with AS, it can play a role in alleviating and treating AS. The active compounds that target VEGFA and AKT1 show strong docking ability and could be potential inhibitors of VEGFA and AKT1. β-carotene has the best docking ability of the bunch, and our study has shown that it can help prevent AS through antioxidants. In short, XFZYD treats AS with multiple components, multiple targets, and multiple pathways, providing ideas and references for AS and the theoretical basis for subsequent experimental studies. But there are also some limitations in our study, there are no further experiments to verify the results. Next, we will verify the results through animal or cells experiments.

## Author contributions

Jingshan Zhao, Lin Liu, Yingyun Li, Boyu Liu performed main analysis and drafted the article. Qing Xu, Quan Shen, Weikang Li helped in the introduction sections. All authors wrote, read, and approved the article.

Conceptualization: Jingshan Zhao

Data curation: Qing Xu, Quan Shen, Weikang Li

Funding acquisition: Jingshan Zhao, Lin Liu

Investigation: Yingyun Li, Lin Liu, Jingshan Zhao, Boyu Liu

Project administration: Jingshan Zhao, Lin Liu

Resources: Yingyun Li, Boyu Liu, Qing Xu, Quan Shen, Weikang Li

Software: Yingyun Li, Quan Shen, Qing Xu

Supervision: Jingshan Zhao, Lin Liu

Validation: Jingshan Zhao, Lin Liu

Writing-original draft: Yingyun Li, Boyu Liu, Jingshan Zhao

Writing-review and editing: Yingyun Li, Lin Liu, Jingshan Zhao
